# Hypomethylation of *Alu* Elements in Post-Menopausal Women with Osteoporosis

**DOI:** 10.1371/journal.pone.0070386

**Published:** 2013-08-21

**Authors:** Pornrutsami Jintaridth, Rungsunn Tungtrongchitr, Sangchai Preutthipan, Apiwat Mutirangura

**Affiliations:** 1 Department of Tropical Nutrition and Food Science, Faculty of Tropical Medicine, Mahidol University Bangkok, Thailand; 2 Department of Obstetrics and Gynecology, Faculty of Medicine, Ramathibodi Hospital, Mahidol University, Thailand; 3 Center for Excellence in Molecular Genetics of Cancer and Human Diseases, Department of Anatomy, Faculty of Medicine, Chulalongkorn University, Bangkok, Thailand; San Francisco Coordinating Center, United States of America

## Abstract

A decrease in genomic methylation commonly occurs in aging cells; however, whether this epigenetic modification leads to age-related phenotypes has not been evaluated. *Alu* elements are the major interspersed repetitive DNA elements in humans that lose DNA methylation in aging individuals. Alu demethylation in blood cells starts at approximately 40 years of age, and the degree of Alu hypomethylation increases with age. Bone mass is lost with aging, particularly in menopausal women with lower body mass. Consequently, osteoporosis is commonly found in thin postmenopausal women. Here, we correlated the Alu methylation level of blood cells with bone density in 323 postmenopausal women. Alu hypomethylation was associated with advanced age and lower bone mass density, (P<0.05). The association between the Alu methylation level and bone mass was independent of age, body mass, and body fat, with an odds ratio [1]  = 0.4316 (0.2087–0.8927). Individuals of the same age with osteopenia, osteoporosis, and a high body mass index have lower Alu methylation levels (P = 0.0005, 0.003, and ≤0.0001, respectively). Finally, when comparing individuals with the same age and body mass, Alu hypomethylation was observed in individuals with lower bone mass (P<0.0001). In conclusion, there are positive correlations between Alu hypomethylation in blood cells and several age-related phenotypes in bone and body fat. Therefore, reduced global methylation may play a role in the systemic senescence process. Further evaluation of Alu hypomethylation may clarify the epigenetic regulation of osteoporosis in post-menopausal women.

## Introduction

Global hypomethylation, the reduction of genome methylation at interspersed repetitive sequences, is an epigenomic alteration that occurs with aging and human diseases, such as cancer and autoimmune diseases. The global hypomethylation pattern in aging is distinct from that observed in some diseases. Methylation levels of Alu elements and long interspersed element-1 s (LINE-1 s) have been evaluated in cancer, autoimmune disease, and aging. Alu and LINE-1 hypomethylation are commonly reported in cancer [2]–[4].(Kitkumthorn N, 2011 #2) Hypomethylation of LINE-1 but not Alu has been reported in systemic lupus erythematosus patients [5]. By contrast, hypomethylation of Alu but not LINE-1 was reported as a global hypomethylation event in aging cells [6]. In this study, we evaluated the association of Alu hypomethylation with age-related phenotypes, focusing on osteoporosis.

Osteoporosis is a common age-related disease. Among individuals older than 50 years of age, half of all women and a quarter of all men break a bone due to osteoporosis [7], [8]. This is most notable in postmenopausal women. The World Health Organization (WHO) characterizes osteoporosis by low bone mass and micro architectural deterioration of bone tissue, which results in an excessively fragile skeleton that is susceptible to fractures [9]–[11]. The most common clinical outcomes are fractures of the spine, hip, and wrist [11]. The prevalence of femoral neck and lumbar spine osteoporosis in 40–80-year-old Thai women in 2000–2001 was 13.6% and 19.8%, respectively [12], [13]. Skeletal size and density develop from early embryogenesis through the intrauterine period, infancy, childhood, and adult life [10]. Genetics and environmental conditions, such as nutrition (calcium and vitamin D), hormones, and lifestyle components, during these all periods contribute to disease burden by impacting low bone mass density (BMD) and modulating on the determination of osteoporotic fractures besides BMD [11], [14]–[16]. BMD is under strong genetic control [11]. Some specific genes involved in all periods of bone development have been shown to be involved in osteoporosis, and the impacts of these genes on osteoporosis are likely to be different among populations with different ethnic backgrounds [10]. Single nucleotide polymorphisms (SNPs) and copy number variations (CNVs) are responsible for a fraction of the genetic component of osteoporosis [10]. Gene-environment interactions are involved in fetal bone development and may cause osteoporosis later in life [1], [10], [17]–[19]. Lifestyle factors associated with low BMD and osteoporosis include smoking, alcohol intake, low calcium intake, vitamin D insufficiency, delayed menarche, low body weight, and physical inactivity [11].

DNA methylation is a possible mechanism of epigenetic regulation of bone development and osteoporosis and plays a significant role in osteogenic cell differentiation and bone metabolism [20]–[22]. *Alu* is the most abundant of the short-interspersed nuclear elements (SINE), with over one million copies per genome. *Alu* elements comprise approximately 11% of the human genome and contain 30% of its methylation sites [23], [24]. Methylation of repetitive elements such as *Alu* is correlated with the total genomic methylation content [23]–[25]. Recently, we demonstrated that a direct correlation between age and a reduction of Alu methylation levels becomes apparent at 34–68 years and that Alu methylation is progressively lost with increased age [6]. Loss of global methylation is associated with genomic instability [26]. Genomic instability is also proposed to be related to the aging process [27]. In addition to overall methylation levels, we recently reported the methylation statuses of LINE-1 loci by classifying the LINE-1 loci into 4 classes: hypermethylated, hypomethylated, and 2 types of partially methylated loci [28]–[31]. We observed that in the presence of certain conditions (smoking), changes in the LINE-1 methylation pattern occurred while the overall LINE-1 methylation level was not significantly changed [30]. Moreover, in some conditions, such as cancer, hypomethylated loci were more sensitive and specific than overall methylation levels [28], [29], [31]. Because osteoporosis is a disease of aging and epigenetic changes are related to age, we were interested in correlating age-related epigenetic variation with bone density. Our aim was to examine whether methylation of Alu is associated with osteoporosis.

## Results

### Age-related phenotypes in correlation with Alu methylation level

In this study, in addition to overall methylation levels, we also classified Alu loci based on the methylation statuses of 2 CpG dinucleotides as hypermethylated loci, mCmC, hypomethylated loci, uCuC, and 2 types of partially methylated, mCuC and uCmC (Fig. S1). The mean and median characteristics of 323 postmenopausal women are described in Table 1 and Table 2. There was a negative correlation between Alu methylation level (%mC) and age and Alu methylation level (%mC) and waist/hip (W/H) ratio and a positive correlation between %mC and the bone mass density (BMD) of many bone regions (Table 1). Using BMD indicator; age, %uCuC and %uCmC were higher and body mass (weight, body mass index (BMI), W/H ratio and %total body fat), systolic blood pressure (SBP), diastolic blood pressure (DBP), spine BMD, hip BMD, radius BMD, bone total BMD, %mC level and %mCmC loci were lower in osteopenia and osteoporosis than normal (Table S1).

**Table 1:** Mean and median of characteristic data and the correlation (r) between %mC and age-related phenotypes among all menopausal subjects.

Characteristics	mean ± SE	median (min-max)	r	N
age (yrs)	57.23±0.39	57.00 (36–75)	−0.13*	323
weight (kg)	56.74±0.53	55.50 (38.1–95)	NS	323
BMI (kg/m^2^)	23.58±0.20	22.97 (15.46–39.54)	NS	321
waist (cm)	78.70±0.51	77.75 (26.0–111)	NS	318
W/H ratio	0.82±0.01	0.81 (0.68–1.05)	−0.12*	336
SBP (mmHg)	124.81±0.77	122.00 (100–168)	NS	253
DBP ((mmHg)	76.55±0.53	80.00 (50–98)	NS	253
total body fat (%)	36.63±0.42	36.90 (19.7–57.90)	NS	236
L1 BMD (g/cm^2^)	0.93±0.01	0.91 (0.42–1.4)	NS	249
L2 BMD (g/cm^2^)	0.98±0.01	0.95 (0.5–1.49)	NS	258
L3 BMD (g/cm^2^)	1.06±0.01	1.03 (0.69–1.57)	NS	258
L4 BMD (g/cm^2^)	1.05±0.01	1.02 (0.71–1.66)	NS	258
L12 BMD (g/cm^2^)	0.96±0.01	0.94 (0.64–1.44)	0.14*	218
L13 BMD (g/cm^2^)	1.02±0.01	0.99 (0.68–1.47)	0.15*	217
L14 BMD (g/cm^2^)	1.02±0.01	0.99 (0.70–1.78)	0.15*	217
L23 BMD (g/cm^2^)	1.03±0.01	1.00 (0.69–1.65)	0.16**	217
L24 BMD (g/cm^2^)	1.04±0.01	1.01 (0.7–1.56)	0.15*	218
L34 BMD (g/cm^2^)	1.06±0.01	1.04 (0.64–1.60)	0.16*	217
femur neck BMD (g/cm^2^)	0.81±0.01	0.79 (0.29–1.67)	NS	252
hip ward BMD (g/cm^2^)	0.65±0.01	0.64 (0.29–1.24)	0.14*	252
femur trochanteric BMD (g/cm^2^)	0.69±0.01	0.68 (0.41–1.07)	NS	252
hip total BMD (g/cm^2^)	0.88±0.01	0.87 (0.23–1.32)	NS	251
radius ud BMD (g/cm^2^)	0.32±0.00	0.31 (0.18–0.52)	NS	284
radius 33 BMD (g/cm^2^)	0.62±0.01	0.63 (0.32–0.82)	NS	285
radius total BMD (g/cm^2^)	0.49±0.00	0.48 (0.26–0.96)	NS	284
bone total BMD (g/cm^2^)	1.08±0.01	1.07 (0.82–1.38)	NS	190

L, spine region lumbar; ± SE, standard error; r, correlation coefficient; n, number of cases;

* *P*<0.05,

** *P*<0.001.

**Table 2:** Mean and median of %mC level, %mCmC loci, %mCuC loci, %uCmC loci, %uCuC loci and %mCuC loci+%uCmC loci among all menopausal subjects.

Alu methylation	mean ± SE	median (min-max)	N
%mC level	30.13±0.30	30.78 (16.71–43.61)	323
%mCmC loci	8.58±0.26	8.06 (0.32–24.22)	323
%mCuC loci	18.00±0.14	17.89 (3.76–25.93)	323
%uCmC loci	25.29±0.24	26.00 (11.76–45.03)	323
%uCuC loci	47.48±0.44	45.93 (21.28–70.85)	323
%mCuC loci+%uCmC loci	43.08±0.27	43.20 (15.98–67.53)	323

± SE, standard error; n, number of cases; %mCmC, %hypermethylated loci;

%uCuC, %hypomethylated loci; %uCmC and %mCuC, %partially methylated loci.

### Age-adjusted correlation between Alu hypomethylation and osteoporosis

**Figure 1:**
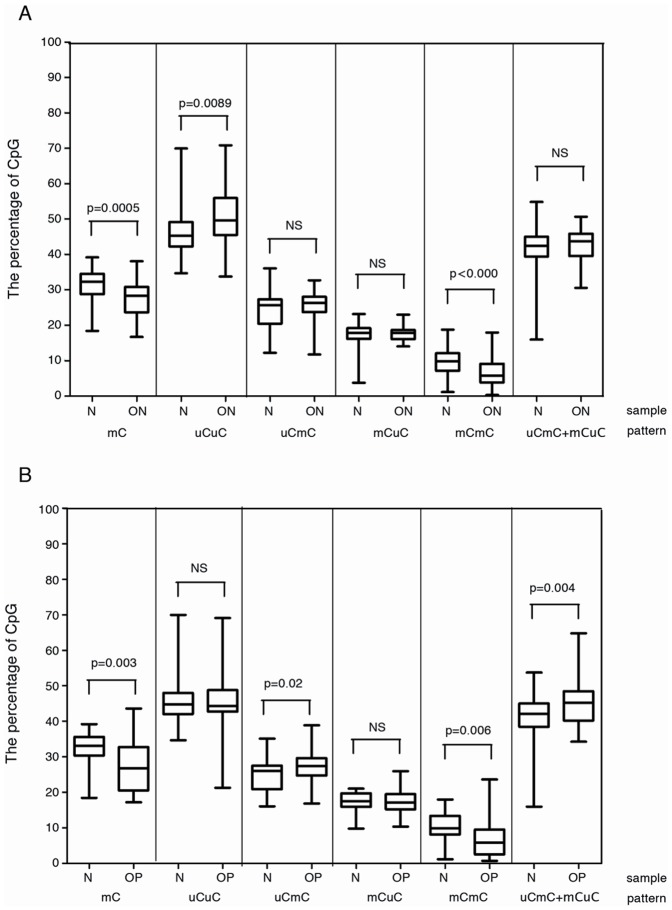
Alu methylation of osteopenia and osteoporosis. (**A**) paired age normal (n = 65) and osteopenia (n = 65); (**B**) paired age normal (n = 35) and osteoporosis (n = 35). N, normal control. ON, osteopenia. OP, osteoporosis. NS, not significant.

To exclude the influence of age on Alu methylation, we paired each osteopenia and osteoporosis individual with a healthy person of the same age. Individuals with osteopenia or osteoporosis had significantly lower %mC than healthy individuals (P≤0.001 and P≤0.001) (Figure 1A&Figure 1B). The hypomethylation level was correlated with a reduction in %mCmC and an increase in %uCuC (Figure 1A&Figure 1B). These data confirmed the correlation between Alu hypomethylation and lower BMD phenotypes; this correlation was not due to age.

### Age-adjusted correlation between Alu hypomethylation and body mass

Subjects with lower body mass (weight, BMI, waist circumference (WC), and %body fat) had significantly lower BMD values (Tables S2 and S3). We examined the correlation between Alu methylation and BMI. High (BMI≥25 kg/m^2^) and low BMI (BMI<25 kg/m^2^) individuals were matched on age.

**Figure 2:**
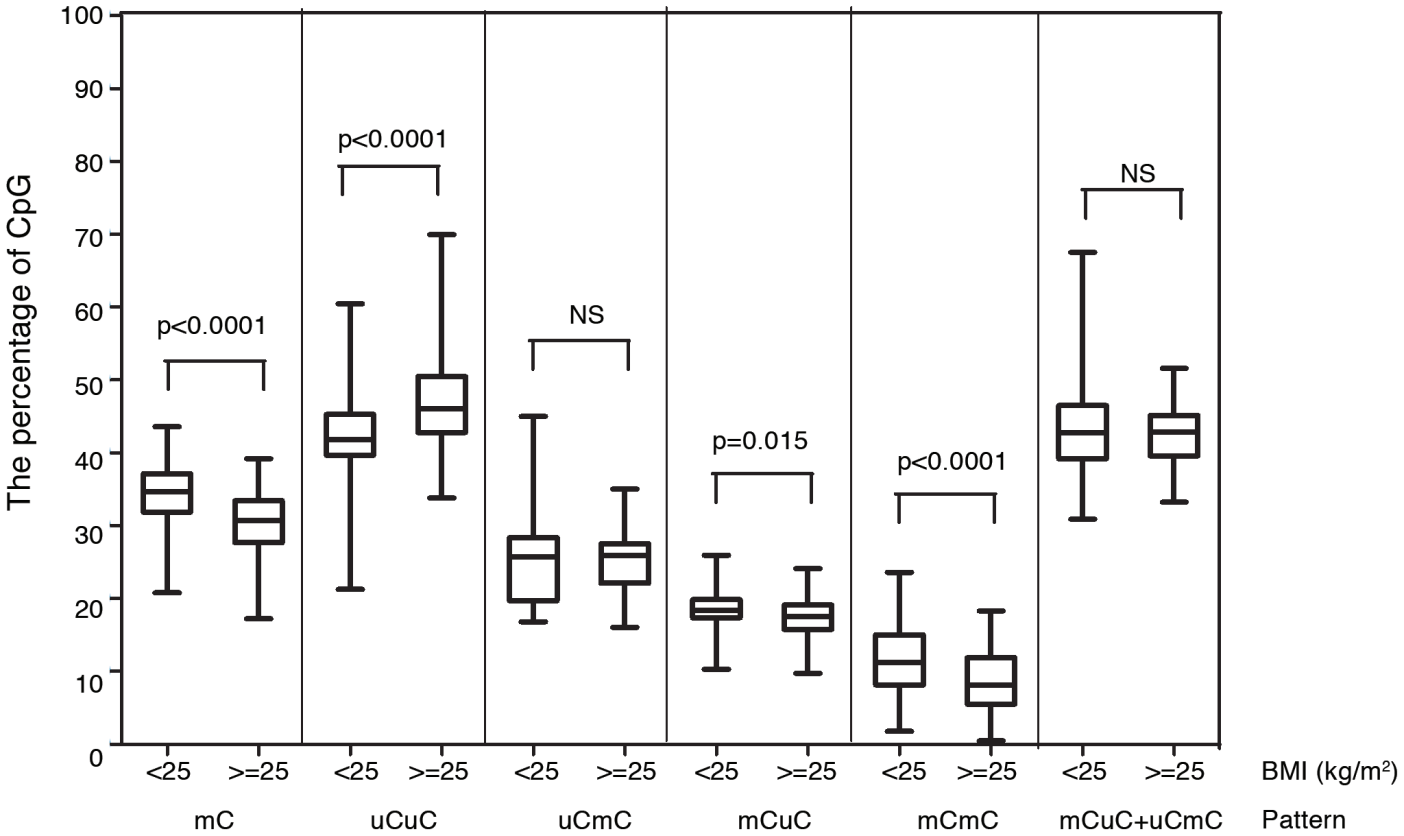
Alu methylation of osteopenia and osteoporosis. Paired age between cases with body mass index (BMI)<25 kg/m^2^ (n = 80) and cases with BMI≥25 kg/m^2^ (n = 80). NS, not significant.

Subjects with a high BMI had significantly lower %mC, %mCmC, and %mCuC than subjects with low BMI (P≤0.001, P≤0.001, and P≤0.05, respectively) but had significantly higher %uCuC (P≤0.001) (Figure 2). Therefore, whereas lower BMI is associated with lower BMD, Alu hypomethylation is directly associated with lower BMD and higher BMI.

### Age and BMI adjusted correlation between Alu hypomethylation and osteoporosis

**Figure 3:**
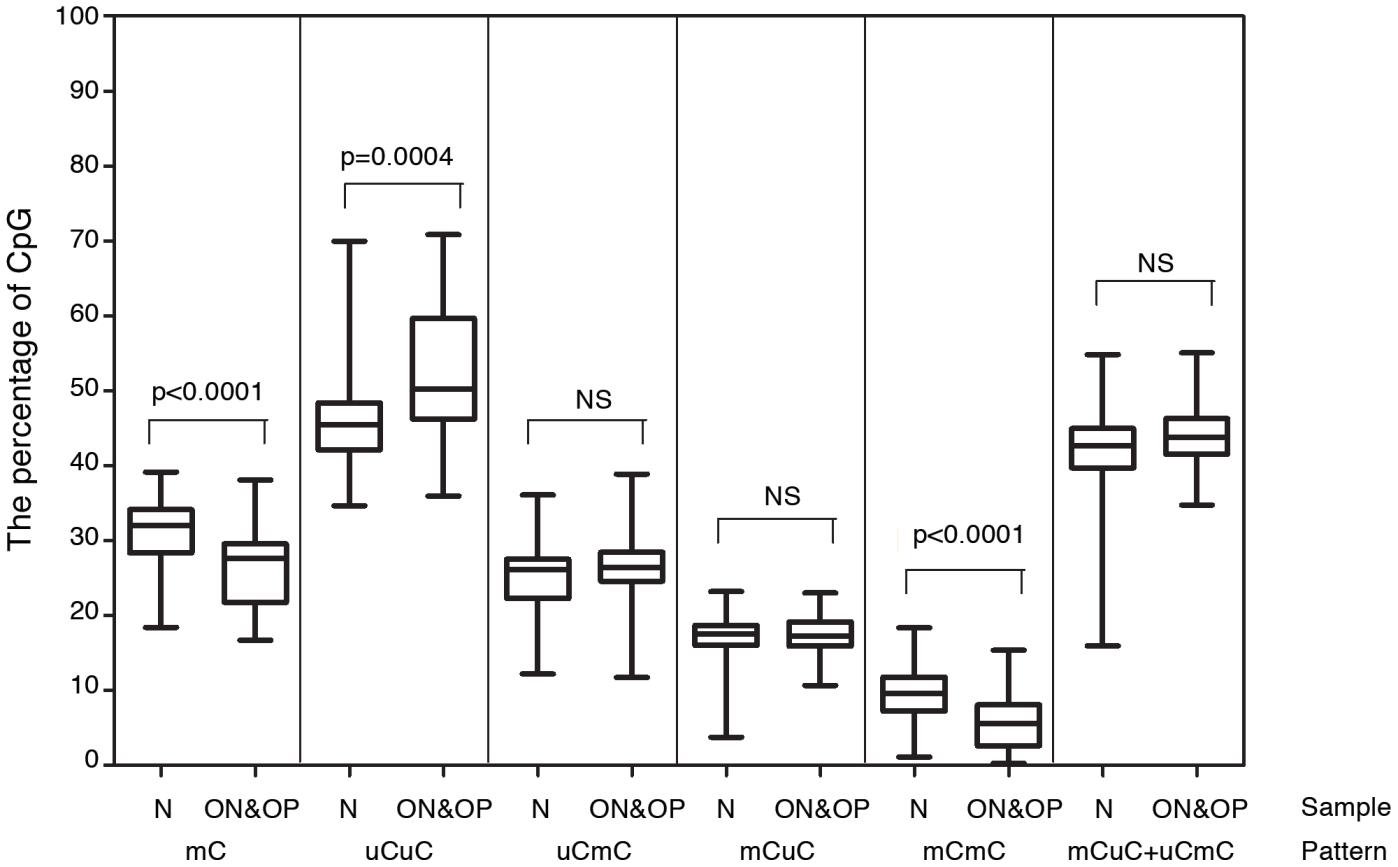
Alu methylation of osteopenia and osteoporosis. Normal (n = 52) and combined osteopenia and osteoporosis (n = 63) were paired by age and BMI.

We further analyzed the association between Alu hypomethylation and osteoporosis by excluding the influences of age and BMI. We matched osteopenia and osteoporosis subjects with controls based on age and BMI. We observed that %mC and %mCmC were significantly lower and %uCuC was significantly higher in the osteopenia and osteoporosis subjects than the controls (P<0.0001 and P<0.0001; and P = 0.0004, respectively) (Figure 3).

### The association between osteoporosis and Alu methylation, age, BMI, and %body fat using logistic regression models

Logistic regression models were used to test the association between several parameters and osteoporosis. Significant associations with age, BMI, %body fat and Alu methylation were confirmed. The %mC (Odds Ratio (OR) = 0.43, P = 0.02), %mCmC (OR = 1.62, P = 0.05), and %uCuC (OR = 0.71, P = 0.04) were considered risk factors for osteoporosis (Table 3). The Alu associations with osteopenia/osteoporosis are independent of age and BMI. These results confirmed the association between Alu hypomethylation in osteoporosis in postmenopausal women.

**Table 3:** Logistic regression analysis when bone mineral density by DEXA (T-score) cut-offs of normal and osteoporosis was the dependent variable and %mC, %mCmC loci, %uCmC loci, %mCuC loci, %uCuC loci, age, BMI, and %body fat were independent variables.

Independent variables	Odds ratio	P-value	95% confidence interval
%mC	0.4316	0.02	0.2087–0.8927
%mCmC loci	1.6172	0.05	0.9988–2.6186
%uCmC loci	1.1121	NS	0.9353–1.3223
%mCuC loci	1.0887	NS	0.8325–1.4236
%uCuC loci	0.7115	0.04	0.5124–0.9879
age	1.2589	0.000	1.1486–1.3797
BMI	0.7030	0.000	0.5922–0.8345
%body fat	0.9998	0.000	0.9997–0.9999

Different levels of significance at *P*≤0.05.

### Bisulfite pyrosequencing analysis

**Figure 4:**
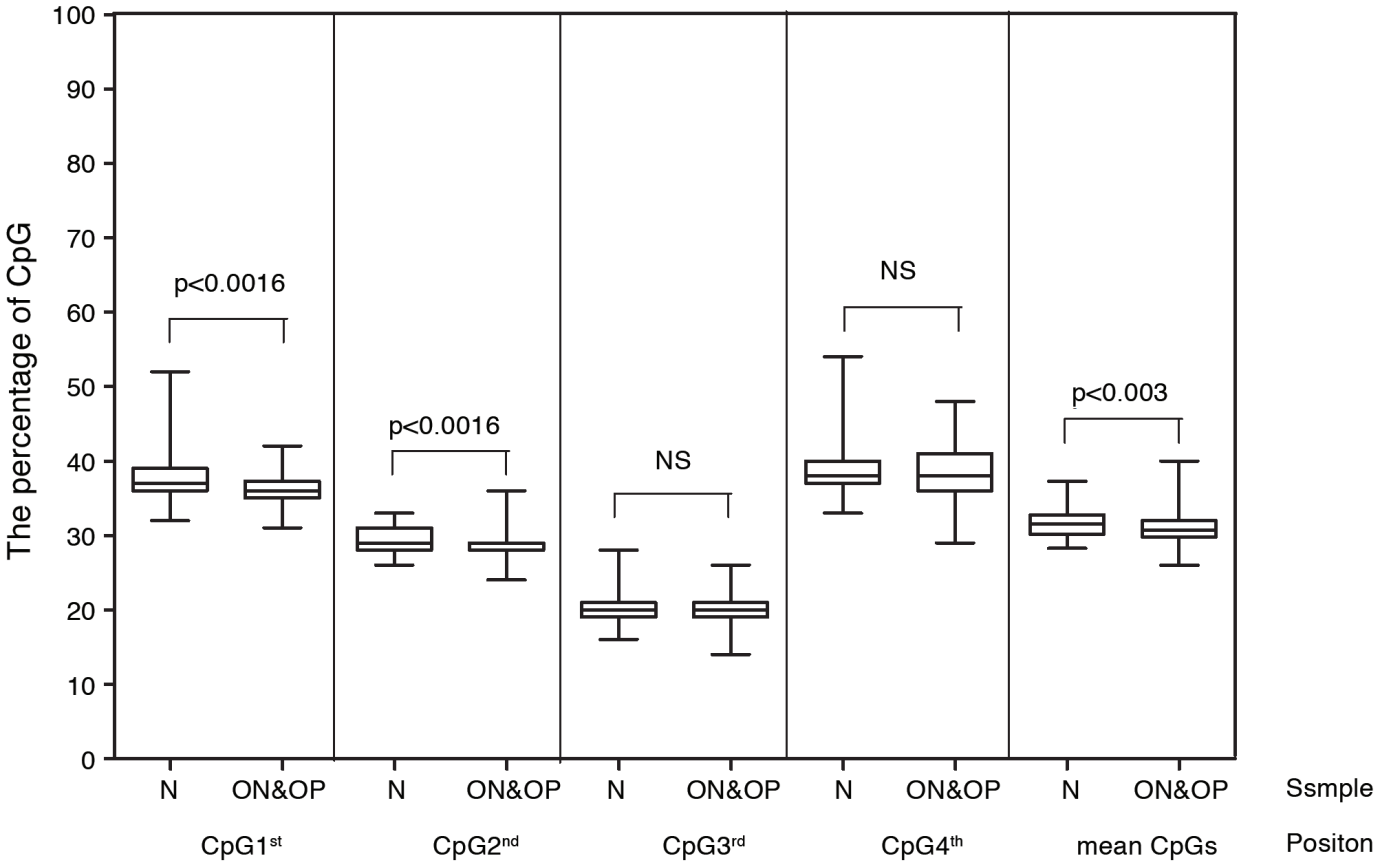
Alu methylation levels by pyrosequencing. 4 CpG and combined methylation levels are observed. N, normal control. ON, osteopenia. OP, osteoporosis.

To study CpG methylation, pyrosequencing analysis was performed on 61 normal, 54 osteopenia, and 31 osteoporosis subjects. The Alu amplicon contains four CpGs, which permits variation in the percentages of methylation (Table S4). Only the 1^st^ and 2^nd^ positions demonstrated lower methylation levels in combined osteopenia and osteoporosis cases (P = 0.0016 and P = 0.0016, respectively) (Figure 4). Overall methylation levels were also lower (P = 0.03) (Figure 4).

## Discussion

Osteoporosis is a common age-related disease that causes low bone mass and micro architectural deterioration of bone tissue, resulting in a fragile skeleton. In this study, we observed lower Alu methylation in peripheral blood cells in subjects with decreased bone mass, increased age, and increased BMI. The data supported that lower Alu methylation levels are directly correlated with aging. This study demonstrates an association but not necessarily causation. Elderly individuals are likely to have lower bone density and consequently develop osteoporosis [32]. They also tend to have higher BMIs [32]. However, individuals with a high BMI are less likely to have osteoporosis because the skeleton responds to mechanical stress, such as body weight, with a stimulation of osteoblast activity and secretion of the bone-protective hormone estrogen from fat tissue [32], [33]. Here, we demonstrated an association between Alu hypomethylation and 2 aging phenotypes (osteoporosis and high BMI), even though the 2 aging phenotypes are inversely correlated.

Analysis of the Alu methylation patterns revealed decreased Alu methylation level, increased hypomethylation loci, decreased hypermethylation loci, and unchanged partial methylation loci. This finding supports the hypothesis that DNA methylation changes in aging may occur in one direction from hypermethylation to hypomethylation or hypermethylation to partial methylation to hypomethylation. This result is in contrast with our previous reports on LINE-1 methylation pattern changes caused by smoking (which are bidirectional) [30].

The mechanism responsible for Alu hypomethylation in the aging process remains unknown. However, our observation suggests that the epigenomic changes may be systemic. While we observed Alu hypomethylation in blood cells in subjects with osteoporosis and increased BMI are phenotypes of bone and fat cells, respectively. Further evaluation of Alu hypomethylation in other cell types will facilitate the elucidation of the mechanisms involved in Alu hypomethylation. Methylation variation can be induced by adverse environmental conditions such as age, gender, race/ethnicity, hormone imbalance, nutritional imbalance, oxidative stress, obese and lifestyle choices [1], [4], [25], [34]–[37]. Only two cellular environments that cause genome-wide hypomethylation have been characterized: malnutrition and increased oxidative stress. Dietary methyl donors such as folic acid, choline, betaine, zinc, vitamin B_12_, vitamin B_6_, and alcohol are precursors of DNA methylation.^33^ In a previous study, methylation donor supplementation resulted in an increase in the number of methylation-variable loci distributed throughout the genome [34]. The precise mechanism for the reduction of genomic methylation by oxidative stress has not yet been described. The generation of hydroxyl radicals causes a wide range of DNA lesions, including base modifications, deletions, strand breakages, and chromosomal rearrangements, that interfere with the ability of DNA to function as a substrate for DNA methyl transferases (DNMTs), resulting in global hypomethylation [38]. ROS production is associated with increased DNA damage and chromosomal degradation, with changes in both hypermethylation and hypomethylation of the DNA [38]. However, an association between global hypomethylation and oxidative stress was recently reported [31], [38], [39]. Interestingly, an association between oxidative stress and osteoporosis as well as age has also been reported [40]–[42]. Therefore, it is reasonable to further explore the connection among aging phenotypes, oxidative stress, and Alu methylation levels.

The significance of Alu hypomethylation in the promotion of aging phenotypes is still unknown. Unlike LINE-1 s, which is important in the regulation of gene expression [43], the role of Alu methylation in controlling gene expression remains to be fully characterized. There are several indications that Alu hypomethylation influences the aging process via genomic instability. First, the loss of DNA methylation in the mouse or human genome leads to genomic instability or increases in mutation rates; by contrast, genomic instability caused by, for example, oxidative stress can lead to global hypomethylation [44]. Second, we reported that histone acetylation, which is associated with DNA hypomethylation [45], spontaneously reduces delayed repair of replication-independent DNA double-stranded breaks and increases γ-H2AX bound DNA [46]. Finally, endogenous γ-H2AX foci are reported to be associated with age-related diseases [47]. Therefore, genomic instability may be one of the underlying mechanisms for the promotion of age-related phenotypes by Alu hypomethylation.

In conclusion, we demonstrated that Alu hypomethylation is associated with aging-associated phenotypes and diseases, increased BMI, decreased bone mass, and osteoporosis. The age-related Alu hypomethylation may be systemic, and Alu methylation levels can be an indicator of age-related phenotypes. Understanding the underlying mechanisms of the correlation between Alu hypomethylation and age-related phenotypes may clarify the roles of epigenetic modifications in age-related disabilities and diseases.

## Materials and Methods

### Subjects

The present analysis is based on 323 menopausal women enrolled in the Menopausal Clinic, Outpatient Department, General Practice Section of the Department of Obstetrics and Gynecology, Ramathibodi Hospital, Bangkok. Subjects were recruited according to the following criteria: each individual 1) agreed to participate and 2) had no history of chronic and/or genetic diseases and/or disorders of bone metabolism. Bone mass density (BMD), which is used as an indicator for osteoporosis diagnosis, was measured for all subjects. The DEXA T-score method of BMD was used: >−1 SD was diagnosed normal, −1 to −2.5 SD was diagnosed with osteopenia, and <−2.5 SD was diagnosed with at least one site of osteoporosis. BMD (g/cm^2^) was assessed at the total body, lumbar spine sites, femur sites, and radius sites, using dual-energy x-ray absorptiometry (Lunar Prodigy®, GE Healthcare, WI, USA). All menopausal women included in this analysis agreed to an assessment of socio-demographic variables and lifestyle risk factors such as smoking and alcohol consumption. Height, weight, and waist circumference (WC) measurements were obtained using standard protocols. The BMI was calculated using the height and weight measurements (weight/[height m^2^]). Percentage of body fat (BF) was measured with an OMRON-bioelectrical impedance analysis instrument.

### Blood collection and DNA preparation for COBRA Alu

Heparinized blood was collected, and DNA was extracted from whole blood using QiAmp DNA blood kits (Qiagen, Hilden, Germany). A total of 200 ng DNA was used for bisulfite treatment. The combined bisulfite restriction analysis (COBRA) consisted of a standard sodium bisulfite PCR treatment followed by restriction digestion and quantitation. Bisulfite modification of genomic DNA was performed using previously published methods [48]. In brief, 200 ng DNA was dissolved in 50 µl distilled water and then denatured in 5.5 µl 2 M NaOH for 10–30 min at 37°C. Next, 30 µl freshly prepared 10 mM hydroquinone and 520 µl 3 M bisulfite (pH 5.0) was added and mixed. The samples were incubated at 50°C for 16 h. The bisulfite-treated DNA was isolated with the Wizard DNA Clean up System. The DNA was eluted with 50 µl of warm water and desulfonated with 5.5 µl of 3 M NaOH for 5 min. The DNA was precipitated (NH_4_OAC-EtOH) using glycogen as a carrier and resuspended in 20 µl water. Bisulfite-treated DNA was stored at −20°C until use.

### COBRA Alu

COBRA is a standard technique for measuring methylation levels of Alu [6], [49]. The primer sequences corresponding to the nucleotides in the regulatory region of the Alu sequence (the Alu Sx subfamily) [23] were F, 5′-GGT GGT TTA MGT TTG TAA TTT TAG TAT TT-3′ and R, 5′-ATT TCA CCA TAT TAA CCA AAC TAA TC-3′. The PCR reactions were performed with the following conditions: 35 cycles of 95°C for 45 sec, 63°C for 45 sec, and 72°C for 45 sec. The PCR products were subsequently digested with 2 U *TaqI* (MBI Fermentas) in TE buffer 3 (Biolab) at 65°C overnight, followed by separation on an 8% nondenaturing polyacrylamide gel. The gel was stained with SyBr Green, and the band intensities were measured with a PhosphoImager using Image Quant software (Molecular Dynamics).

### Genomic Alu methylation distribution pattern and methylation analysis

A schematic representation and example of the COBRA of the Alu distribution patterns is shown in Figure 1. For COBRA Alu, the Alu loci of each individual were divided into 4 groups depending on the methylation status of the 2 CpG sites as follows: 1) hypermethylated loci: mCmC, complete methylation at both CpG sites, yielded three fragments of 37, 32, and 29 bp; 2) hypomethylated loci: uCuC, fully unmethylated sequence, yielded 1 uncut amplicon with a size of 98 bp; and 3) and 4) partially methylated loci: uCmC and mCuC. The unmethylated 37 bp CpG and methylated 69 bp CpG sequence yielded 2 fragments of 69 and 29 bp. By contrast, the methylated 37 bp CpG and unmethylated 69 bp CpG sequence yielded 2 fragments of 37 and 61 bp (Figure S1A). To improve and expand the applications of Alu methylation, we determined the Alu methylation profile using the Alu methylation distribution pattern combined with the percentage of methylation. Each product was analyzed as a percentage of product intensity divided by the double strand length. The % number of hypermethylated loci (%mCmC) was %32/30, of hypomethylated loci (%uCuC) was %98/98, of partially methylated loci (%uCmC) was %69/68, and of partially methylated loci (%mCuC) was %61/60.

### Bisulfite pyrosequencing Alu analysis

For the pyrosequencing Alu analysis, bisulfite modification of genomic DNA was prepared using the previously mentioned COBRA method. Alu methylation was quantitated by pyrosequencing with the following primers and conditions: 20 µM forward primer 5′-GGT GGT TTA MGT TTG TAA TTT TAG TAT TT-3′ and 20 µM reverse biotinylated primer 5′-ATT TCA CCA TAT TAA CCA AAC TAA TC-3′. The purified, single-stranded biotinylated PCR products acted as a template for annealing of the first pyrosequencing primer, 5′GTTTGTAATTTTAGTATTTTGG-3′, to detect CpG in the first 3 positions of the Alu amplicon and of the second pyrosequencing primer, 5′GATTATTTGAGGTTAGGAGT-3′, to detect CpG in the last 4 positions of the Alu amplicon (0.3 µM final concentration). The PCR products were then subjected to sequencing using an automatically generated nucleotide dispensation order for the sequences to be analyzed. The Pyrograms were analyzed using CpG mode to determine the proportion of C/T and thus the proportion of methylated and unmethylated cytosines at the targeted positions. The degree of methylation was evaluated at 4 CpG methylation sites.

### Statistical analysis

Data were analyzed with SPSS statistical software version 11.5. The averages and distributions of the characteristic data of menopausal women are shown as the mean±SD and median. Pearson's correlation between the Alu methylation pattern and the characteristic data of the population were used to determine 95% confidence intervals of association. The DEXA T-score was used to divide subjects into 3 groups: normal, osteopenia, and osteoporosis; and the average of the characteristic data of each group are shown as the mean±SD. A T-test was used to determine the differences at a P-value≤0.05 between the groups in the matched cases based on age. A T-test was used to determine the differences of BMD at each site, and Alu methylation pattern at a P-value≤0.05 between the groups in the matched cases divided by indicator BMI (≥25 vs <25 kg/m^2^). Logistic regression models were used to evaluate the association between indicator BMD by DEXA T-score as a dependent variable specified as a binary outcome, normal and osteoporosis, and %mC, %mCmC loci, %uCmC loci, %mCuC loci, %uCuC loci, age, BMI, and %body fat as independent predictors. All the independent variables entered into the model at the same time.

### Ethics statement

The study protocol and procedures were approved by the Ethical Committee of the Faculty of Tropical Medicine, Mahidol University and the faculty of Medicine, Ramathibodi Hospital. All study participants gave written informed consent.

## Supporting Information

Figure S1 **Combined bisulfite restriction analysis: Alu sequence of sodium bisulfite treated whole blood DNA of menopausal subjects.** (A) Alu methylation pattern by COBRA PCR. The Alu amplicons were 98 bp and contained 2 CpG dinucleotides. After digestion with the enzyme Taq1, which targets the methylated cytosine site, the Alu sequences were divided into 4 product fragments depending on the methylation status of the 2 CpG sites as follows: 1) hypermethylated loci: mCmC; 2) hypomethylated loci: uCuC; and 3) and 4) partially methylated loci: uCmC and mCuC. (B) COBRA Alu in menopausal subjects DNA treated Taq 1 enzyme, Alu sequences with complete methylation at both CpG sites yielded 3 fragments of 37, 32, 29 bp, while the fully unmethylated sequence yielded 1 fragment, 98 bp uncut amplicon. For the partially methylated sequences, unmethylated 37 bp CpG and methylated 69 bp CpG yielded 2 fragments, 69 and 29 bp. By contrast, methylated 37 bp CpG and unmethylated 69 bp CpG yielded 2 fragments, 37 and 61 bp.
(RAR)

Table S1 **Mean and median of age, BMI, waist, SBP, DBP, %total 1 body fat, BMD and Alu methylation pattern among normal, osteopenia, and osteoporosis diagnosed by DEXA T-score (>−1.0 SD = normal, −1.0 to −2.5 SD = osteopenia and <−2.5 SD = osteoporosis).**
(PDF)

Table S2 **Mean difference (X ± SE) of weight, BMI, waist, systolic 1 blood pressure (SBP), diastolic blood pressure (DBP), and %total body fat between normal and osteopenia and between normal and osteoporosis as diagnosed by DEXA T-score (>−1.0 SD = normal, −1.0 to −2.5 SD = osteopenia and <−2.5 SD = osteoporosis) in the matched cases.** Normal and osteopenia and normal and osteoporosis were paired by age.
(PDF)

Table S3 **Mean difference (X ± SE) of bone mass density (BMD) at each 1 region: L1, L2, L3, L4, L1–2, L1–3, L1–4, L2–3, L2–4, L3–4, femur neck, hip ward, femur trochanteric, hip total, radius ud, radius 33, radius total, and bone total between matched cases with body mass index (BMI)<25 kg/m^2^ and BMI>25 kg/m^2^.** Paired age between cases with body mass index (BMI)<25 kg/m^2^ and cases with BMI>25 kg/m^2^.
(PDF)

Table S4 **The percentages of Alu methylation levels in each position 1 of CpG and mean of all CpG in normal, osteopenia, and osteoporosis cases.**
(PDF)
