# Supplementary material for: Hypomethylation of Alu Elements in Post-Menopausal Women with Osteoporosis
Source: PLoS One. 2013 Aug 21;8(8):e70386. doi: 10.1371/journal.pone.0070386 (PMC3749148; doi:10.1371/journal.pone.0070386)
Supplement: Table S1 — Mean and median of age, BMI, waist, SBP, DBP, %total 1 body fat, BMD and Alu methylation pattern among normal, osteopenia, and osteoporosis diagnosed by DEXA T-score (>−1.0 SD = normal, −1.0 to −2.5 SD = osteopenia and <−2.5 SD = osteoporosis). (PDF) [file pone.0070386.s002.pdf]

Table S1 Mean and median of age, BMI, waist, SBP, DBP, %total body fat, BMD and Alu methylation pattern among normal, osteopenia, and osteoporosis diagnosed by DEXA T-score (>-1.0 SD=normal, -1.0 to –2.5 SD=osteopenia and <-2.5 SD=osteoporosis)

Characteristics	normal (76 cases)	osteopenia (156 cases)	osteoporosis (91 cases)
age (yrs)	52.80±0.73	57.07±0.49	61.56±0.59
weight (kg)	61.71±1.70	56.94±0.72	51.72±0.79
BMI (kg/m^2)	25.42±0.42	23.68±0.24	21.76±0.28
waist (cm)	82.56±1.11	78.44±0.74	75.94±0.75
SBP (mmHg)	127.2±1.78	124.83±1.11	123.17±1.36
DBP ((mmHg)	78.45±1.27	76.55±0.78	75.26±0.86
total body fat (%)	37.57±1.14	37.30±0.52	35.49±0.69
L1 BMD (g/cm^2)	1.11±0.02	0.97±0.01	0.83±0.01
L2 BMD (g/cm^2)	1.20±0.02	1.00±0.01	0.87±0.01
L3 BMD (g/cm^2)	1.30±0.02	1.08±0.01	0.94±0.01
L4 BMD (g/cm^2)	1.30±0.03	1.08±0.01	0.94±0.01
L12 BMD (g/cm^2)	1.13±0.04	0.98±0.01	0.86±0.01
L13 BMD (g/cm^2)	1.19±0.03	1.02±0.01	0.89±0.01
L14 BMD (g/cm^2)	1.21±0.04	1.04±0.01	0.91±0.01
L23 BMD (g/cm^2)	1.25±0.03	1.04±0.01	0.92±0.01
L24 BMD (g/cm^2)	1.25±0.03	1.06±0.01	0.93±0.01
L34 BMD (g/cm^2)	1.27±0.04	1.08±0.01	0.94±0.01
femur neck BMD (g/cm^2)	1.00±0.02	0.82±0.01	0.73±0.01
hip ward BMD (g/cm^2)	0.91±0.03	0.68±0.01	0.56±0.01
femur trochanteric BMD (g/cm^2)	0.87±0.02	0.73±0.01	0.62±0.01
hip total BMD (g/cm^2)	1.09±0.02	0.91±0.01	0.80±0.01
radius ud BMD (g/cm^2)	0.38±0.01	0.32±0.00	0.26±0.00
radius 33 BMD (g/cm^2)	0.71±0.01	0.63±0.01	0.55±0.01
radius total BMD (g/cm^2)	0.56±0.01	0.48±0.00	0.42±0.01
bone total BMD (g/cm^2)	1.18±0.12	1.09±0.06	1.00±0.01
%mC	30.60±0.55	30.57±0.44	28.99±0.59
%mCmC	9.21±0.49	9.04±0.39	7.27±0.45
%uCmC	24.69±0.49	24.83±0.36	26.56±0.47
%mCuC	17.46±0.34	17.93±0.17	17.87±0.29
%uCuC	47.38±0.81	47.10±0.60	48.22±0.95
