# Supplementary material for: Hypomethylation of Alu Elements in Post-Menopausal Women with Osteoporosis
Source: PLoS One. 2013 Aug 21;8(8):e70386. doi: 10.1371/journal.pone.0070386 (PMC3749148; doi:10.1371/journal.pone.0070386)
Supplement: Table S2 — Mean difference (X ± SE) of weight, BMI, waist, systolic 1 blood pressure (SBP), diastolic blood pressure (DBP), and %total body fat between normal and osteopenia and between normal and osteoporosis as diagnosed by DEXA T-score (>−1.0 SD = normal, −1.0 to −2.5 SD = osteopenia and <−2.5 SD = osteoporosis) in the matched cases. Normal and osteopenia and normal and osteoporosis were paired by age. (PDF) [file pone.0070386.s003.pdf]

Table S2 Mean difference (X±SE) of weight, BMI, waist, systolic blood pressure (SBP), diastolic blood pressure (DBP), and % total body fat between normal and osteopenia and between normal and osteoporosis as diagnosed by DEXA T-score (>-1.0 SD=normal, -1.0 to -2.5 SD=osteopenia and <-2.5 SD=osteoporosis) in the matched cases. Normal and osteopenia and normal and osteoporosis were paired by age

Variables	Subjects					
	**normal**	**osteopenia**	**p-value[a]**	**normal**	**osteoporosis**	**p-value[b]**
weight (kg)	62.06±1.34	56.85±0.94	0.002	62.67±1.92	51.43±1.29	0.000
BMI (kg/m^2)	25.74±0.52	23.74±0.42	0.003	25.95±0.69	21.69±0.49	0.000
waist (cm)	83.14±1.61	78.62±1.07	0.006	83.14±1.61	75.06±1.30	0.000
SBP (mmHg)	128.47±1.84	123.48±1.31	0.03	129.36±2.93	120.85±1.66	0.02
DBP(mmHg)	79.42±1.28	76.12±1.17	NS	78.73±1.88	75.52±1.26	NS
%total body fat	38.85±1.36	36.69±0.79	NS	40.02±1.85	35.42±1.25	0.04

[a]Significant differences by T-test between normal and osteopenia at P-value≤0.05

[b]Significant differences by T-test between normal and osteoporosis at P-value≤0.05
