# Supplementary material for: Hypomethylation of Alu Elements in Post-Menopausal Women with Osteoporosis
Source: PLoS One. 2013 Aug 21;8(8):e70386. doi: 10.1371/journal.pone.0070386 (PMC3749148; doi:10.1371/journal.pone.0070386)
Supplement: Table S3 — Mean difference (X ± SE) of bone mass density (BMD) at each 1 region: L1, L2, L3, L4, L1–2, L1–3, L1–4, L2–3, L2–4, L3–4, femur neck, hip ward, femur trochanteric, hip total, radius ud, radius 33, radius total, and bone total between matched cases with body mass index (BMI)<25 kg/m2 and BMI>25 kg/m2. Paired age between cases with body mass index (BMI)<25 kg/m2 and cases with BMI>25 kg/m2. (PDF) [file pone.0070386.s004.pdf]

Table S3 Mean difference (X±SE) of bone mass density (BMD) at each region: L1, L2, L3, L4, L1-2, L1-3, L1-4, L2-3, L2-4, L3-4, femur neck, hip ward, femur trochanteric, hip total, radius ud, radius 33, radius total, and bone total between matched cases with body mass index (BMI)<25 kg/m^2 and BMI$\geq$25 kg/m^2. Paired age between cases with body mass index (BMI)<25 kg/m^2 and cases with BMI$\geq$25 kg/m^2

**BMD (g/cm^2)**	**BMI (kg/m^2)**		**P-value[a]**
	**<25 (kg/m^2)**	**$\geq$25 (kg/m^2)**	
L1 BMD	0.89±0.02	0.98±0.02	0.000
L2 BMD	0.94±0.02	1.04±0.02	0.000
L3 BMD	1.03±0.02	1.12±0.02	0.004
L4 BMD	1.03±0.02	1.14±0.02	0.001
L1-2 BMD	0.94±0.02	1.04±0.02	0.001
L1-3 BMD	0.98±0.02	1.08±0.02	0.001
L1-4 BMD	1.00±0.02	1.11±0.02	0.001
L2-3 BMD	1.01±0.02	1.12±0.03	0.001
L2-4 BMD	1.02±0.02	1.13±0.02	0.001
L3-4 BMD	1.04±0.03	1.16±0.02	0.001
femur neck BMD	0.77±0.01	0.85±0.01	0.001
hip ward BMD	0.63±0.02	0.70±0.02	0.009
femur trochanteric BMD	0.67±0.02	0.75±0.01	0.000
hip total BMD	0.86±0.01	0.96±0.02	0.000
radius ud BMD	0.31±0.01	0.35±0.01	0.000
radius 33 BMDl	0.61±0.01	0.64±0.01	0.034
radius total BMD	0.48±0.01	0.52±0.01	0.001
bone total BMD	1.07±0.01	1.11±0.01	0.020

[a]Significant differences by T-test between BMI<25 kg/m^2 and BMI≥25 kg/m^2 at P-value≤0.05
