# Supplementary material for: Hypomethylation of Alu Elements in Post-Menopausal Women with Osteoporosis
Source: PLoS One. 2013 Aug 21;8(8):e70386. doi: 10.1371/journal.pone.0070386 (PMC3749148; doi:10.1371/journal.pone.0070386)
Supplement: Table S4 — The percentages of Alu methylation levels in each position 1 of CpG and mean of all CpG in normal, osteopenia, and osteoporosis cases. (PDF) [file pone.0070386.s005.pdf]

Table S4 The percentages of Alu methylation levels in each position of CpG and mean of all CpG in normal, osteopenia, and osteoporosis cases.

**cases**	**CpG Alu methylation**				
	**CpG position 1**	**CpG position 2**	**CpG position 3**	**CpG position 4**	**all CpG mean**
**normal**	38.01±0.48	29.37±0.23	20.41±0.30	38.57±0.44	31.59±0.25
**osteopenia**	36.80±0.27	28.59±0.30	20.22±0.21	38.15±0.48	31.01±0.27
**osteoporosis**	35.71±0.37	28.29±0.28	20.15±0.30	38.55±0.64	30.67±0.29
